# Stenting in Palliation of Unresectable Esophageal Cancer

**DOI:** 10.1007/s00268-018-4722-7

**Published:** 2018-06-26

**Authors:** Janusz R Włodarczyk, Jarosław Kużdżał

**Affiliations:** 1 Department of Thoracic and Surgical Oncology, Jagiellonian University Collegium Medicum, John Paul II Hospital, Kraków, Poland; 2 Department of Thoracic Surgery, John Paul II Hospital, ul. Prądnicka 80, 31-202 Kraków, Poland

## Abstract

**Background:**

The aim of this study was to analyze the safety and effectiveness of stenting using partially covered self-expandable stents in palliation of dysphagia in patients with unresectable esophageal cancer.

**Methods:**

Retrospective analysis of hospital records of all patients who underwent esophageal stenting in the period 2008–2015 was performed. The study included patients with unresectable esophageal and esophagogastric cancer.

**Results:**

There were 442 patients included. Mean age was 56 years (range 28–89), and 379 were males. In 40 (9.0%) patients, stenting was performed in the cervical, in 150 (39.3%)—in the middle thoracic, in 141 (31.9%)—in lower thoracic esophagus and in 111 (25.1%)—in the esophagogastric junction. Stenting resulted in significant alleviation of dysphagia grade (3.0 vs. 1.0, *p* = 0.00001). During the follow-up, 55 (12.4%) patients experienced recurrent dysphagia due to tumor or granulation tissue overgrowth, and in 18 (4.1%) patients, migration of the stent occurred, for which an independent risk factor was adjuvant chemo- and/or radiation therapy (*p* = 0.001). Minor complications included chest pain (54.5%), delayed complete stent expansion (12.0%), feeling of a foreign body (25.3%), hiccup (1.6%), gastroesophageal reflux (45.6%) and post-discharge pneumonia (2.5%). A feeling of a foreign body in the esophagus was significantly more common after stenting of the cervical esophagus (*p* = 0.0001), and hiccup was more common after stenting of the esophagogastric junction (*p* = 0.02). Major complications included bleeding (1.3%), respiratory insufficiency (0.7%), esophageal perforation (0.9%) and irregular heartburn (2.3%). Overall procedure-related mortality was 0.4%. The median survival time was 117.8 days (range 2–732).

**Conclusions:**

Stenting is an effective procedure in relieving dysphagia in patients with unresectable malignant esophageal stenosis and is associated with low rate of postoperative and long-term complications.

## Introduction

Squamous cell carcinoma of the esophagus is the eighth most frequent cancer in the world, whereas carcinoma of esophagogastric junction (OGJ) is the tumor with the highest dynamics of incidence in the last two decades [1–4].

It is estimated that in North America there are 5–10 cases of esophageal cancer per 100,000 inhabitants; however, depending on the geographical area, the incidence may increase up to 100/100,000, such as in Iran [5, 6].

Multimodal treatment consisting of preoperative chemoradiation therapy followed by complete resection and lymphadenectomy is a standard in therapeutic management. However, curative-intent treatment is possible in only 20–40% of patients. In a majority of them, palliative management is the only option, due to local advancement and/or distant metastases [7]. In such cases, options of palliative treatment include: chemo- and/or radiation therapy, brachytherapy, stenting, laser ablation and photodynamic therapy. Among them, stenting has a unique advantage of immediate relief of dysphagia. Although stenting is a safe and effective way to relieve the symptoms of dysphagia and to improve the comfort of life, it is not free from side effects, which may occur in the early and late period after its implantation. Among the side effects, mild symptoms can be distinguished, which do not require intervention, but also life-threatening and fatal ones [8, 9].

In this study, we present one of the largest retrospective analyses with prospective follow-up of patients with esophageal cancer, who underwent esophageal stenting due to unresectability of the tumor or medical inoperability. The aim of this study was to evaluate the safety and efficacy of stenting in patients with esophageal squamous cell carcinoma and carcinoma of the esophagogastric junction, complications, re-interventions and survival after the treatment.

## Materials and methods

This retrospective analysis of hospital records included data of a consecutive group of patients with advanced esophageal carcinoma, treated between 2008 and 2015 in Department of Thoracic Surgery, Jagiellonian University Collegium Medicum. Demographic and clinical data including age, sex, weight, dysphagia, dyspnea, chemotherapy/chemoradiation, technical success rate, stent migration, complications and survival were evaluated.

### Inclusion criteria

The study included all patients treated in the period 2008–2015 for unresectable or medically inoperable esophageal or (OGJ) cancer, regardless of histological type.

### Exclusion criteria

Exclusion criteria included:

- Preterminal condition, Karnofsky score ≤ 40%;
- Patients with mediastinal infiltration causing dysphagia in the course of lung cancer, lymphomas and other malignancies.

### Pre-treatment assessment

Unresectability was determined on the basis of chest radiography, abdominal ultrasound, computed tomography (CT) of the chest and the upper abdomen, positron-emission tomography (PET) and endoscopy, with the endoscopic ultrasound (EUS) and endobronchial ultrasound (EBUS). Disease staging was based on the UICC classification [10]. Dysphagia was assessed according to a four-grade scale [11]:

- 0—no dysphagia;
- 1—swallowing of a semiliquid diet;
- 2—swallowing of a liquid diet;
- 3—dysphagia to the liquids and saliva.

Patients diagnosed with fistula in the course of esophageal or bronchogenic cancer were classified into four groups according to fistula location [12]:

- Type 1—fistula to the mediastinum;
- Type 2—fistula to the trachea;
- Type 3—fistula to the bronchus;
- Type 4—fistula after stenting.

Dyspnea severity was assessed with a four-grade scale [12]:

- 0—less than 30% of tracheal or/and bronchial stenosis, no dyspnea;
- 1—30–50% stenosis, dyspnea upon exercise;
- 2—50–70% stenosis, dyspnea during daily activities;
- 3—more than 70% stenosis, dyspnea while resting.

Patient performance status was assessed according to Karnofsky score [13].

### Intervention

Esophageal stenting was performed under general anesthesia. Location of the stenosis was endoscopically identified, and in case of narrow stenosis, dilatation was performed with Savary–Gilliard dilators, up to the size of 10 Fr. After the dilatation, the neoplastic infiltration length was assessed using a small-diameter endoscope, then a guidewire was inserted and the esophageal stent was introduced over it. Deployment of the stent was performed under endoscopic control. Partially covered self-expandable metallic stents (70, 90 or 120 mm long and 18 mm diameter, Ultraflex, Boston Scientific, Natick, MA, USA) were used.

Double stenting was performed in patients with unresectable esophageal cancer involving the airway, with dysphagia and dyspnea;

- Airway compression or infiltration posing the risk of severe airway compromise after expansion of the esophageal stent.

As a rule, airway stenting was performed before esophageal stenting. The double stenting procedure was performed under general anesthesia. The self-expandable Ultraflex stents (Boston Scientific, Natick, MA, USA) were used for stenting of fistulas to the trachea and silicone Y stents (Demed, Mikołów, Poland) in case of fistula located in the tracheal bifurcation and main bronchi. Stenting with the use of silicone Y stents was performed using the Freitag forceps according to the technique described elsewhere [14].

### Complications

Peri-, intra- and postoperative complications and any additional procedures were recorded. Complications after stenting were classified as minor or major. Minor complications were defined as those subsiding spontaneously or following pharmacological treatment only, potentially requiring endoscopy. All other, including life threatening or fatal, were defined as major complications.

Postoperative complications were defined as early (occurring within ≤ 30 days following stenting) or late (occurring later than >  30 days following stenting).

### Follow-up

Following the procedure, patients received a liquid diet the same day. Routinely, on the first day after the procedure a follow-up chest radiogram was obtained and dyspnea and dysphagia scores were assessed. Patients received detailed instructions regarding nutrition at discharge from the hospital. Patients were followed up every 30 days thereafter. If the follow-up visit in the clinic was not feasible, patients were interviewed by phone. During the follow-up visit, the patients’ general condition, dysphagia and dyspnea were assessed.

### Statistical analysis

Statistical analysis was performed using the STATISTICA 11 PL software package (StatSoft, Tulsa Oklahoma, USA). The Mann–Whitney test was used to compare two samples. Kruskal–Wallis test was used to compare three or more attempts. In order to evaluate the changes over time (dysphagia before and after stenting, migration), the Wilcoxon signed rank test was applied. To assess the significance of connections between data on nominal scale, the Fisher’s Chi-square test or Fisher’s exact test was used. The logistic regression model was used to find the risk factors for complications after stenting. If an important factor was found, odds ratio (OR) was calculated along with 95% confidence interval. Survival was calculated using Kaplan–Meier method. Gehena–Wilcoxon test was used to compare survival curves. *p* < 0.05 was considered as statistically significant.

## Results

### Characteristics of the study group

**Fig. 1:**
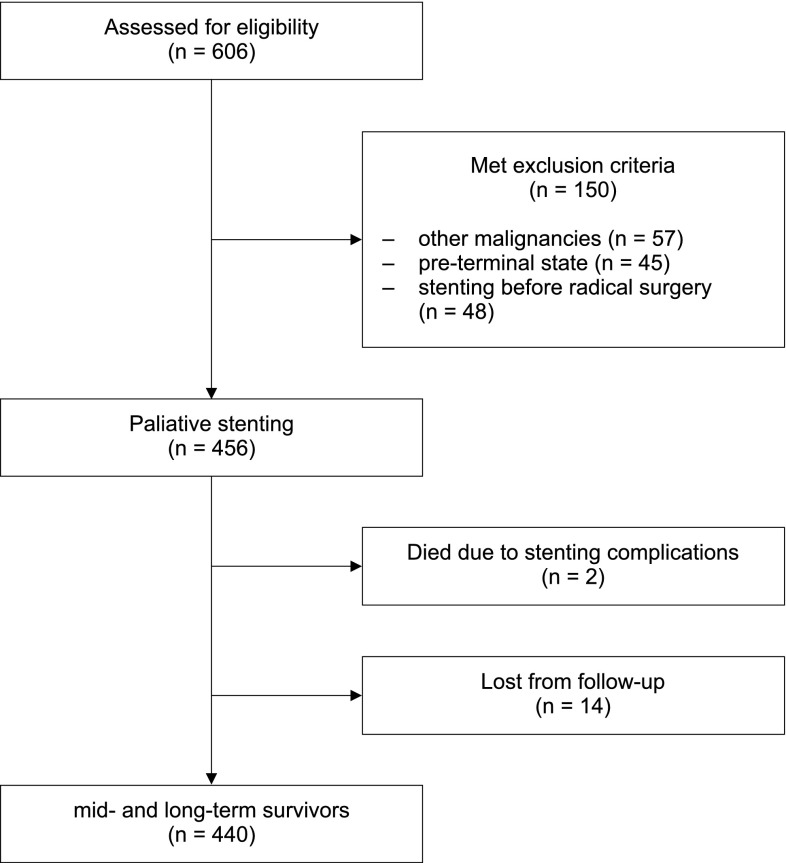
Patients’ flowchart

Between 2008 and 2015, 606 patients underwent esophageal stenting for malignant esophageal obstruction. The flowchart of the study is presented in Fig. 1. One hundred and sixty-four patients who met the exclusion criteria were excluded from this analysis, including:

- 46 patients with lung cancer;
- 2 patients with thyroid cancer;
- 1 patient with colorectal cancer;
- 1 patient with breast cancer;
- 7 patients with lymphomas;
- 45 due to the preterminal state or Karnofsky score ≤ 40%;
- 14 patients who were lost from postoperative follow-up;
- 48 patients stented before planned surgical resection.

The final analysis included homogenous group of 442 patients with esophageal or OEJ cancer, who underwent esophageal stenting procedure.

Patients presented with body weight loss from 4 to 40 kg, dysphagia, cough and cachexia. The mean length of neoplastic infiltration in the esophagus was 5.9 cm (range 4–12 cm).

In 40 (9.0%) patients, stenting of the upper segment of the esophagus was performed, including:

- 28 (6.3%) and 30 (6.34%) patients with the tumor located between 18 and 21 cm from the incisors;
- 12 (2.7%) and 18 (4.16%) patients with the tumor located between 22 and 25 cm from the incisors.

In 150 (39.3%) patients, stenting was performed in the middle part of the esophagus, in 141 (31.9%)—in the lower thoracic part of the esophagus and in 111 (25.1%)—in the OGJ.

Nineteen (4.3%) patients had primary fistula to the mediastinum or the airway. Fifteen (3.04%) patients with fistula developed after the stenting procedure. Adjuvant chemo- and radiation therapy was administered to 201 (45.5%) patients (Table 1).

**Table 1:** Demographic and clinical characteristics of the study group

Characteristics of the study population	Number of patients (no/%)
Sex (male/female)	442 (379/63)
Mean age (range), (years)	59 (35–82)
Histopathology	
SCC	331 (74.9%)
OGJ	111 (25.1%)
Location	
Upper esophagus	40 (9.1%)
Middle esophagus	150 (33.9%)
Lower esophagus	141 (31.9%)
Esophagogastric junction	111 (25.1%)
Dysphagia score	
Grade 1	0
Grade 2	389 (88.0%)
Grade 3	53 (12.0%)
Esophago-airway fistula (OAF)	34 (7.7%)
Type 1	7 (1.6%)
Type 2	4 (0.9%)
Type 3	8 (1.8%)
Type 4	15 (3.4%)
Treatment after stenting	201 (45.5)
CTH	51 (11.5)
RTH	17 (3.8)
CTH/RTH	131 (29.6)
BTH	2 (0.4)
Median survival time (range)	117.8 (2–732)
SCC	131.2 (2–732)
OGJ cancer	109.8 (38–221)
OAF	74.5 (41–432)

*CTH* chemotherapy, *RTH* radiotherapy, *CTH/RTH* chemo and radiotherapy, *BTH* brachytherapy, *OAF* esophago-airway fistula, *SCC* squamous cell carcinoma, *OGJ* esophagogastric junction

#### Technical success

Stenting procedure could not be performed in 3 (0.6%) patients due to complete obstruction of the esophagus. These patients underwent laparotomy and gastrostomy. Thus, the technical success rate was 99.4%.

#### Dysphagia relief

After stenting procedure, swallowing improvement was observed in all the patients. The mean dysphagia score improved from 3.0 (range 2–3) before stenting to 1 (range 1–2) after the stenting procedure (*p* = 0.00001).

### Early complications

#### Minor complications

After esophageal stenting, 241 (54.5%) patients reported chest pain: in 28 (6.3%) patients with stent in the proximal esophagus, in 94 (21.2%)—in the middle part, in 78 (17.6%)—in the lower part and in 49 (11.0%)—in the EGJ. Pain occurred more frequently in patients with stents the proximal and middle part of the esophagus (*p* = 0.004). In 209 (42.3%) patients, mild analgesia was required and the pain subsided within 2–4 days after the procedure, whereas in 33 (7.5%) patients long-term analgetic medication was needed.

Incomplete immediate stent expansion occurred in 53 (12.0%) patients. In all of them, the stent expanded fully without any intervention within 48 h.

Difficulties in swallowing, associated with the feeling of a foreign body, were reported by 112 (25.3%) patients, including 29 (6.6%) with a stent in the proximal esophagus, 30 (6.8%)—in the middle part, in 21 (4.8%)—in the lower part and in 29 (5.7%) of them after the stenting of OGJ. The feeling of a foreign body was present only in patients with stents in the proximal part of the esophagus (*p* = 0.0001). These symptoms subsided completely or partially within 3–7 days.

Hiccup occurred in 7 (1.6%) patients after esophageal stenting: In 4 of them, it happened after stenting of the EGJ and required stent removal in 3 cases, and in 3 (0.7%) patients after stenting of the lower part of the thoracic esophagus (*p* = 0.02), requiring stent removal in 1 (0.2%) patient. In 3 patients with squamous cell carcinoma and in 4 with OGJ, carcinoma early migration of the stent occurred. Two hundred and two (45.6%) patients reported reflux symptoms, and they required treatment with proton-pump inhibitors. After discharge from the hospital, in 11 (2.5%) patients pneumonia occurred and they received outpatient treatment.

#### Major complications

Immediately after the stenting, bleeding occurred in 6 (1.3%) patients, and in 3 (0.7%) of them, transfusion of 2–4 units of packed red blood cells was necessary.

Irregular heartburn occurred in 10 (2.3%) patients (Table 2). Symptoms of respiratory insufficiency requiring mechanical ventilation for 2–4 days occurred in 3 (0.7%) patients. In all these patients, respiratory function improved and ventilatory support was discontinued. In 4 (0.9%) patients, perforation of the esophageal wall occurred during the pre-stenting dilatation. In 2 (0.4%) patients, it happened in the middle part and in 2 (0.4%) patients in the lower part of the esophagus. All these patients were treated conservatively. Three of them (0.7%) recovered, and one (0.2%) patient died. Another patient died directly after the stenting due to heart arrhythmia not responding neither to pharmacological nor to electrical therapy. Thus, the overall procedure-related mortality was 0.4%.

**Table 2:** Complications of stenting

Complication after stenting	SCC	OGJ	*p* value
Migration	10 (2.0%)	8 (1.8%)	
Partial—no re-stenting	1 (0.2%)	1 (0.2%)	
Complete (no re-stenting)	1 (0.2%)	0	
Complete (re-stenting)	8 (1.8%)	7 (1.42%)	0.06
Restenosis	39 (7.92%)	16 (3.6%)	
Granulation—proximal end of the stent	32 (7.2%)	14 (3.1%)	
Granulation—distal end of the stent	5 (1.1%)	2 (0.4%)	
Malignant obstruction	2 (0.4%)	0	0.54
Re-stenting	45 (10.1%)	23 (5.2%)	
Stent removal and re-stenting	16 (3.6%)	10 (2.2%)	
Telescope stenting	15 (3.4%)	4 (0.9%)	
Re-stenting with one stent	14 (3.1%)	9 (2.0%)	
Airway stenosis	4 (0.9%)	0	
Critical—airway stenting	2 (0.4%)	0	
Non-critical—observation	2 (0.4%)	0	
Perforation	3 (0.7%)	0	
OAF	34 (7.7%)	0	
Primary	19 (4.3%		
After stenting	15 (3.4%)		
Respiratory failure	3 (0.7%)	0	
Death	2 (0.4%)	0	

*OAF* esophago-airway fistula, *SCC* squamous cell carcinoma, *OGJ* esophagogastric junction

### Late complications

#### Re-interventions

In 18 (4.1%) patients, migration of the stent occurred. It happened in the middle thoracic part of the esophagus in 3 (0.7%) the patients, in the lower part in 7 (1.6%) patients and in 8 (1.8%) patients in the OGJ (*p* = 0.06) (Table 2). Adjuvant treatment with CTH and/or RTH was a risk factor for stent migration [*p* = 0.001; OR 6.08 (95% CI 2.01–5.83)]. There were no significant differences in the migration rates when SCC was compared with adenocarcinoma of the OGJ (*p* = 0.06).

In 7 patients with squamous cell carcinoma and in 4 with OGJ carcinoma, late migration of the stent occurred.

Dysphagia associated with stent obliteration by the ingrowing granulation tissue occurred in 55 (12.4%) patients: in 46 (10.4%) at the proximal end of stent and in 9 (2.0%) patients at the distal end. In 2 (0.4%) patients, ingrowing tumor was observed. Development of granulation tissue was observed in the period from 27 to 103 days (mean 72 days) since stent implantation. In 5 (1.1%) patients, ingrowing granulation tissue was observed in the proximal part of the esophagus, in 16 (3.6%) patients in the middle part, in 19 (4.3%) patients in the lower part and in 16 (3.6%) patients in the EGJ (*p* = 0.54). In patients with stent obliteration by tumor or granulation tissue ingrowth, restoration of patency was performed with the use of argon plasma coagulation followed by re-stenting.

Stent removal and re-stenting procedure were required in 28 (6.3%) and 31 (6.3%) patients, respectively. Forty-six (10.4%) patients with stent obstruction received CTH and/or RTH, which was a risk factor for stent obstruction [*p* = 0.00006; OR 3.42 (95% CI 2.01–5.83)].

In 4 (0.9%) patients, esophageal stenting resulted in compression of the airway. In 2 (0.4%) of them, symptoms of dyspnea required additional stenting of the bronchial tree with Y stent; in the remaining 2 (0.4%) patients, compression of the bronchial tree < 30% of lumen occurred, without symptoms of dyspnea and not requiring additional stenting. These patients were subjected to follow-up (Table 3).

**Table 3:** Interventional management

Complication after stenting	SCC	OGJ	Secondary	*p* value
Migration	10 (2.03%)	8 (1.62%)	2 (0.4%)	
Partial—no re-stenting	1 (0.2%)	1 (0.2%)		
Complete—no re-stenting	0	0	1 (1.42%)	
Complete—re-stenting	9 (1.82%)	7 (1.6%)(1.42%)	1 (1.42%)	*p* = 0.6
Restenosis	39 (7.92%)	16 (3.25%)	4 (0.81%)	
Granulation—proximal end of the stent	32 (6.5%)	14 (2.84%)	3 (3.4%)	
Granulation—distal end of the stent	5 (1.01%)	2 (0.4%)	1 (1.41%)	
Malignant obstruction	2 (0.4%)	0		*p* = 0.54
Re-stenting	52 (10.56%)	17 (3.45%)		
Stent removal	22 (4.47%)	9 (1.82%)		
Telescope stenting	30 (6.09%)	8 (1.62%)		
Airway stenosis	10 (2.03%)			
Critical—airway stenting	6 (1.21%)			
Non-critical—observation	4 (0.81%)			
Perforation	3 (0.6%)			
OAF	15 (3.04%)			
Respiratory failure	3 (0.6%)			
Death	2 (0.4%)			

*OAF* esophago-airway fistula, *SCC* squamous cell carcinoma, *OGJ* esophagogastric junction

### Survival

**Fig. 2:**
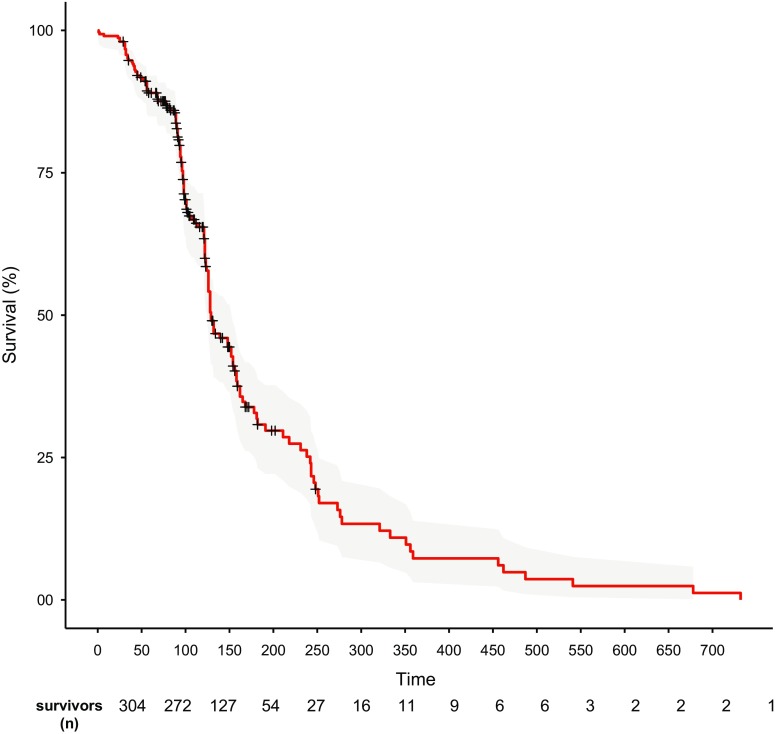
Survival curve of patients with unresectable esophageal carcinoma

The follow-up period ranged between 1 and 732 days. Median survival time was 117.8 days (range 2–732) (Fig. 2). Median survival time was longer in patients with SCC than with adenocarcinoma: 158 (range 2–732) versus 110 (range 38-221) days (*p* = 0.06). Median survival time in patients with OAF was 74.5 days (range 41–432).

### Esophago-airway fistula

Esophago-airway fistula (OAF) was found in 34 (7.7%) patients (Table 1). Nineteen (4.3%) patients had OAF at presentation, and in 15 patients, it developed after stenting. Among all 34 patients with OAF, it developed in 26 (5.9%) patients after double stenting (esophagus and airway) and in 8 patients after esophageal stenting only. In all those patients, improvement in dysphagia score (2.81 vs. 1.3, *p* = 0.0001 and 2.68 vs. 1.0, *p* = 0.0001), dyspnea score (2.85 vs. 0.36, *p* = 0.001 and 1.69 vs. 0.08, *p* = 0.0001) and Karnofsky score (59 vs. 70, *p* = 0.0001) was achieved. There was no significant improvement in BMI (18.48 vs. 18.39, *p* = 0.6). The median survival after stenting of the OAF was 74.5 days (range 41–432) days. The survival did not correlate with the use of chemotherapy or chemoradiation (*p* = 0.54).

## Discussion

A primitive stent was used for the first time for intubation of the esophageal stenosis in 1885, and rapid development of stenting occurred together with the development of endoscopy [5, 15]. In the 1970s and 1980s, rigid and semirigid stents were used, and since the 1990s, non-covered, partially covered and fully covered self-expandable stents have been available. Despite technological advancements and simplicity of implantation, their use is not free from life-threatening complications [8, 16, 17]. Stenting in inoperable esophageal cancer is an attractive alternative to surgical gastro-jejunostomy, enabling oral nutrition, improvement in metabolic status and comfort of life, but also allowing complementary treatment (chemo and/or chemo-radiotherapy). It is a relatively simple procedure; however, due to the anatomical relationship of the esophagus to the bronchial tree, possible complications and re-interventions after stenting, this treatment should be planned in reference centers.

One of the most frequent complications of esophageal stenting is ingrowth or overgrowth of granulation tissue or tumor at the ends of the stent. In our study, the percentage of tissue overgrowth was 11.99%, with the applied stenting margin of 4 cm. These results are consistent with the literature data, showing its incidence of 4–47% (higher in cases of the use of non-covered stents) [17, 18]. Results are much better if covered stents are used: Ingrowth of granulation tissue can occur only at the uncovered ends of the stent. Reportedly, it was observed in 3–18% of cases [19–22] which, again, corresponds with our results. The mechanism of overgrowth of granulation tissue is not precisely known, but according to some authors it grows more slowly in stents with lumen diameter of 18 mm [23]. This was, however, not confirmed in other studies using stents with wide diameter of 23 mm [24, 25].

The second most frequent complication is stent migration, which reportedly occurs in 0–20% cases [21–23]. Our results confirm the finding of chemotherapy or chemoradiation as an independent risk factor for migration. In the randomized study on stenting in unresectable gastric cancer, Lee et al. observed statistically significant differences in migration and obstruction for uncovered stents compared to covered stents (9.5 vs. 5.4 and 7.1 vs. 37.8%) [26]. Van den Berg et al. [27] performed stenting procedures of OAF and of postoperative anastomotic leaks using the 23 mm stents and did not confirm their superiority regarding risk of migration. In a randomized study, Siersma et al. demonstrated that 12 out of 13 migrations were associated with smaller stent diameter and only one was associated with larger diameter of the stent [28]. As we only used 18-mm-wide stents, we could not analyze the impact of stent diameter on the risk of migration. On the other hand, the use of stents of larger diameter can be associated with a higher rate of perforations and bleeding [23]. Sgourakis et al. [29] did not observe an advantage of any kind of stents with regard to complications and reflux, even in cases of application of stents with antireflux valves. Based on the analysis of a group of 332 patients, Park et al. observed that stenting of an obstruction present in the area of EGJ and the administration of adjuvant chemoradiation therapy were independent risk factors for migration [30]. Also, the stents with anti-migration mechanism (SX Ella, Niti S, Alimaax) do not always prevent migration effectively [19, 31]. In our series, we did not find significant difference in migration rate regarding location of the stent.

Although esophageal stenting is characterized by a very high technical success rate, it is associated with the risk of life-threatening complications. One of them is development of an OAF following the procedure. According to the literature, it occurs in up to 10% of patients after the stenting procedure. Ferreira et al. reported the occurrence of such fistulas in 7 out of 126 treated patients and Uitdehaag et al. in 2 out of 44 patients, after the application of SX Ella stents with anti-migration mechanism [20, 32]. In our group, OAF occurred after stenting in 15 (3.4%) patients.

Other most frequent severe complications include: hemorrhage, which occurs in 2–28%, perforations, perioperative mortality, which was estimated to be 0.5–7%, and a 30-day mortality that ranges from 7 to 18% [20, 23, 32–37]. In our series, bleeding occurred in 6 (1.3%) patients, perforation in 3 (0.7%), perioperative mortality in 2 (0.4%) and 30-day mortality in 3 (0.7%). These results compare favorably with the literature data.

Esophageal stenting may enable the introduction of chemotherapy or chemoradiation. Chemotherapy enables relief of dysphagia and full oral nutrition, so the European Society of Gastrointestinal Endoscopy recommends its administration [38, 39]. There is no clear statement whether it should be introduced before the stenting or after it. Also, adjuvant therapy is associated with risk of life-threatening complications [38–40]. Yakami et al. reported a case of death as a result of fistula between the esophagus and the left atrium, which occurred after radiation therapy. The author suggests the application of stenting procedure in patients who do not respond to chemotherapy or chemoradiation [41]. Park et al. reported that the administration of adjuvant therapy is an independent risk factor for the occurrence of stent migration and obstruction [30]. In our series, chemotherapy or chemoradiation was associated with higher risk factor for stent migration and stent obstruction.

Generally, the use of partially covered self-expandable metallic stents in inoperable/unresectable esophageal cancer is safe and effective palliative procedure, with low rate of complications and perioperative mortality.

## References

[1] Surveillance, epidemiology, and end results program turning (2013) SEER stat fact sheets: esophageal cancer. Available from: http://seer.cancer.gov/statfacts/html/esoph.html Retrieved 9 Nov 2013

[2] Khushalani NI (2008). Cancer of the esophagus and stomach. Mayo Clin Proc..

[3] Ferlay J, Shin HR, Bray F (2010). Estimates of worldwide burden of cancer in 2008: GLOBOCAN 2008. Int J Cancer.

[4] Sehdev A, Catencci DV (2013). Gastroesophageal cancer: focus on epidemiology, classification, and staging. Discov Med.

[5] Brian G, Denittis A, Christopher GRW, Halprin EC, Ferez CA, Brady L (2008). Esophageal cancer. Perez and Brady’s principles and practice of radiation oncology.

[6] Aledavood A, Anvari K, Sabouri G (2011). Esophageal cancer in Northeast of Iran. Iran J Cancer Prev.

[7] Stein HJ, Siewert JR (2004). Improved prognosis of resected esophageal cancer. World J Surg.

[8] Raijman I, Siddique I, Ajani J (1998). Palliation of malignant dysphagia and fistulae with coated expandable metal stents: experience with 101 patients. Gastrointest Endosc.

[9] Shaikh M, Choudhury NR, Knott R (2015). Engineering stent based delivery system for esophageal cancer using docetaxel. Mol Pharm.

[10] UICC-International Union Against Cancer (2009). TNM classification of malignant tumours.

[11] Blazeby JM, Williams MH, Brookes ST (1995). Quality of life measurement in patients with esophageal cancer. Gut.

[12] Włodarczyk J, Kużdżał J (2016). Double stenting for malignant esophago-respiratory fistula. Videosurgery Miniinv.

[13] Karnofsky DA, Abelmann WH, Craver LF (1948). The use of nitrogen mustards in the palliative treatment of cancer. With particular reference to bronchogenic carcinoma. Cancer.

[14] Stephens KE, Wood DE (2000). Bronchoscopic management of central airway obstruction. J Thorac Cardiovasc Surg.

[15] Fugger R, Niederle B, Jantsch H (1990). Endoscopic tube implantation for palliation of malignant stenosis. Endoscopy.

[16] Domschke W, Foerster EC, Matek W (1990). Self-expanding mesh stent for esophageal cancer stenosis. Endoscopy.

[17] Mayoral W, Fleischer D, Salcedo J (2000). Nonmalignant obstruction is a common problem with metal stents in the treatment of esophageal cancer. Gastrointest Endosc.

[18] Homs MY, Steyerberg EW, Eijkenboom WM (2004). Single dose brachytherapy versus metal stent placement for the palliation of dysphagia from esophageal cancer: multicenter randomized trial. Lancet.

[19] Conio M, Repici A, Battaglia G (2007). A randomized prospective comparison of self expandable plastic stents and partially covered self expandable metal stents in the palliation of malignant esophageal dysphagia. Am J Gastroenterol.

[20] Uitdehaag MJ, Siersema PD, Spaander MC (2010). A new fully covered stent with antimigration properties for the palliation of malignant dysphagia: a prospective cohort study. Gastrointest Endosc.

[21] Vakil N, Morris AI, Marcon N (2001). A prospective, randomized, controlled trial of covered expandable metal stents in the palliation of malignant esophageal obstruction at the gastroesophageal junction. Am J Gastroenterol.

[22] Golder M, Tekkis PP, Kennedy C, Lath S (2001). Chest pain following esophageal stenting for malignant dysphagia. Clin Radiol.

[23] Verschuur EM, Steyerberg EW, Kuipers EJ (2007). Effect of stent size on complications and recurrent dysphagia in patients with esophageal or gastric cardia cancer. Gastrointest Endosc.

[24] Bassi M, Luigiano C, Fabbri C (2015). Large diameter fully covered self-expanding metal stent placement for palliation of proximal malignant esophageal strictures. Dis Esophagus.

[25] Tahiri M, Ferraro P, Duranceau A (2015). Self-expanding metallic stent placement with an exaggerated 5-cm proximal tumor covering for palliation of esophageal cancer. Ann Gastroenterol.

[26] Lee H, Min BH, Lee JH (2015). Covered Metallic Stents with an anti-migration design vs. uncovered stents for the palliation of malignant gastric outlet obstruction: a Multicenter, randomized trial. Am J Gastroenterol.

[27] van den Berg MW, Kerbert AC, van Soest EJ (2016). Safety and efficacy of a fully covered large-diameter self-expanding metal stent for the treatment of upper gastrointestinal perforations, anastomotic leaks, and fistula. Dis Esophagus.

[28] Siersema PD, Hop WC, van Blankenstein M (2001). A comparison of 3 types of covered metal stents for the palliation of patients caused by esophagogastric carcinoma: a prospective, randomized study. Gastrointest Endosc.

[29] Sgourakis G, Gockel I, Radtke A (2010). The use of self-expanding stents in esophageal and gastroesophageal junction cancer palliation: a meta analysis and meta-regression analysis of outcomes. Dig Dis Sci.

[30] Park JH, Song HY, Shin JH (2015). Migration of retrievable expandable metallic stents inserted for malignant esophageal strictures: incidence, management, and prognostic factors in 332 patients. AJR Am J Roentgenol.

[31] Conigliaro R, Battaglia G, Repici A (2007). Polyflex stents for malignant esophageal and esophago-gastric stricture: a prospective, multicenter study. Eur J Gastroenterol Hepatol.

[32] Ferreira F, Bastos P, Ribeiro A (2012). A comparative study between fluoroscopic and endoscopic guidance in palliative esophageal stent placement. Dis Esophagus.

[33] Spaander MC, Baron TH, Siersema PD (2016). Esophageal stenting for benign and malignant disease: ESGE clinical guideline. Endoscopy.

[34] Madeya S, Borsch G (1992). Upper intestinal endoscopy in 188 bronchial cancer patients and 118 breast cancer patients with abdominal symptoms. Med Klin.

[35] Lopes CV, Pesenti Ch, Bories E, Caillol F, Giovannini M (2008). Self expiable metallic stents for palliative treatment of digestive cancer. Clin Gastroenterol.

[36] Wilkes EA, Jackson IM, Cole AT (2007). Insertion of expandable metallic stents in esophageal cancer without fluoroscopy in safe and effective: a 5-year experience. Gastrointest Endosc.

[37] Verschuur EM, Homs MY, Steyerberg EW (2006). A new esophageal stent design (Niti-S stent) for the prevention of migration: a prospective study in 42 patients. Gastrointest Endosc.

[38] Ross WA, Alkassab F, Lynch PM (2007). Evolving role of self-expanding metal stents in the treatment of malignant dysphagia and fistulas. Gastrointest Endosc.

[39] Ohtsu A, Boku N, Muro K (1999). Definitive chemoradiotherapy for T4 and/or M1 lymph node squamous cell carcinoma of the esophagus. J Clin Oncol.

[40] Touchefeu Y, Archambeaud I, Landi B (2014). Chemotherapy versus self-expanding metal stent as primary treatment of severe dysphagia from unresectable esophageal or gastro-esophageal junction cancer. Dig Liver Dis.

[41] Yakami M, Mitsumori M, Sai H (2003). Development of severe complications caused by stent placement followed by definitive radiation therapy for T4 esophageal cancer. Int J Clin Oncol.

